# Influence of *Serratia marcescens* and *Rhodococcus rhodnii* on the Humoral Immunity of *Rhodnius prolixus*

**DOI:** 10.3390/ijms222010901

**Published:** 2021-10-09

**Authors:** Kate K. S. Batista, Cecília S. Vieira, Marcela B. Figueiredo, Samara G. Costa-Latgé, Patrícia Azambuja, Fernando A. Genta, Daniele P. Castro

**Affiliations:** 1 Laboratório de Bioquímica e Fisiologia de Insetos, Instituto Oswaldo Cruz (IOC/Fiocruz), Rio de Janeiro 21040-360, Brazil; kateksbatista@gmail.com (K.K.S.B.); ceciliastahl@gmail.com (C.S.V.); samara.latge@ioc.fiocruz.br (S.G.C.-L.); genta@ioc.fiocruz.br (F.A.G.); 2 Institute of Life Science, Swansea University Medical School, Swansea SA2 8PP, UK; marcela.figueiredo@gmail.com; 3 Programa de Pós-Graduação em Ciências e Biotecnologia, Universidade Federal Fluminense, Niteroi 24210-201, Brazil; azambuja.p@gmail.com; 4 Departamento de Entomologia Molecular, Instituto Nacional de Entomologia Molecular (INCT-EM), Rio de Janeiro 21941-599, Brazil

**Keywords:** insect immunity, triatomine, microbiota, bacteria, insect physiology, antibiotic, aposimbiotic

## Abstract

Chagas disease is a human infectious disease caused by *Trypanosoma cruzi* and can be transmitted by triatomine vectors, such as *Rhodnius prolixus*. One limiting factor for *T. cruzi* development is the composition of the bacterial gut microbiota in the triatomine. Herein, we analyzed the humoral immune responses of *R. prolixus* nymphs treated with antibiotics and subsequently recolonized with either *Serratia marcescens* or *Rhodococcus rhodnii*. The treatment with antibiotics reduced the bacterial load in the digestive tract, and the recolonization with each bacterium was successfully detected seven days after treatment. The antibiotic-treated insects, recolonized with *S. marcescens*, presented reduced antibacterial activity against *Staphylococcus aureus* and phenoloxidase activity in hemolymph, and lower nitric oxide synthase (NOS) and higher defensin C gene (DefC) gene expression in the fat body. These insects also presented a higher expression of DefC, lower prolixicin (Prol), and lower NOS levels in the anterior midgut. However, the antibiotic-treated insects recolonized with *R. rhodnii* had increased antibacterial activity against *Escherichia coli* and lower activity against *S. aureus*, higher phenoloxidase activity in hemolymph, and lower NOS expression in the fat body. In the anterior midgut, these insects presented higher *NOS*, defensin A (DefA) and DefC expression, and lower Prol expression. The *R. prolixus* immune modulation by these two bacteria was observed not only in the midgut, but also systemically in the fat body, and may be crucial for the development and transmission of the parasites *Trypanosoma cruzi* and *Trypanosoma rangeli*.

## 1. Introduction

Chagas disease was described by Carlos Chagas in 1909 [1] and is considered a severe public health problem, being one of 21 neglected diseases listed by the World Health Organization. The etiologic agent of Chagas disease, the parasite *Trypanosoma cruzi*, is transmitted by species of the subfamily Triatominae (Hemiptera, Reduviidae). It is estimated that about ten thousand human deaths occur yearly due to complications linked to Chagas disease. About six to seven million people are infected globally, and particularly in Latin America, an endemic region. However, in recent decades, the infection has also been detected in the United States, Canada, and in several European countries [2]. 

The main triatomine species responsible for *T. cruzi* transmission are *Triatoma brasiliensis*, *Panstrongylus megistus*, and *Rhodnius prolixus* [3,4] These vectors are hematophagous insects that can acquire the parasite while feeding in infected mammal reservoirs [4,5].

The complete development of *T. cruzi* depends on the success of the multiplication of epimastigotes along the midgut of the vector, which might be affected by diverse factors mainly related to the parasite strain, physiological aspects of the triatomine species, and the intestinal microbiota [6,7]. The differentiation of *T. cruzi* into infective forms takes place in the insect rectum [8,9]. Transmission can occur through the deposition of the infective forms eliminated with the insect feces or urine near the bite in the host, or by the contamination of food with infected feces, which is considered oral transmission [4,5,10].

The co-evolution between the parasite, the vector, and its microbiota can result in a specific tripartite interaction. A specific parasite strain can modulate the insect immune system and affect the gut microbiota. At the same time, the commensal bacteria can stimulate immune priming in the insect gut, protecting it from further parasite infection [11,12,13]. The microbiota is also fundamental for insect physiology, favoring insect digestion, and priming the immune system [11,12,13,14,15,16]. In this sense, the gut bacterial microbiota impacts the development of trypanosomatids in insects [17,18,19]. In *R. prolixus*, the intestinal microbiota components identified to date are *Serratia*, *Dietzia*, *Gordonia*, *Mycobacterium*, *Corynebacterium*, *Rhodococcus*, *Pectobacterium*, and *Staphylococcus* [17,20,21,22,23,24]. However, knowledge of the interaction between the triatomine and the *T. cruzi* with the bacterial species *Rhodococcus rhodnii* and *Serratia marcescens* is still scarce [17,18,23].

*Rhodococcus rhodnii* is a Gram-positive bacterium belonging to the Nocardiaceae family. It was first observed in *R. prolixus* in 1926 by Duncan [25]. Wigglesworth described the same bacteria in the gut of *R. prolixus*, *Triatoma rubrofasciata*, *Triatoma infestans*, and *Triatoma flavida* [26]. It has been shown that egg surfaces and adult feces transmit *R. rhodnii* to the gut epithelium of the newborn insect. Subsequently, several authors have demonstrated the close relationship between *R. rhodnii* and *R. prolixus*. Aposymbiotic nymphs, free of *R. rhodnii* [27,28] and nymphs fed with rabbit blood immunized against *R. rhodnii* [29], did not reach adulthood. The symbiotic relationship between *R. rhodnii* and *R. prolixus* in the digestive tract was also suggested by supplementing aposymbiotic nymphs with B-complex vitamins, which allowed insect ecdysis, as described by Lake and Friend [30]. The ability of *R. rhodnii* for vitamin B synthesis was recently confirmed by the annotation of its genome [31]. A recent work demonstrated that not only *R. rhodnii* can supply B-complex vitamins to *R. prolixus*, but also different microbes from *Rhodnius* microbiota are able to produce vitamin B derivatives, due to the fact that they have the necessary genes required for the biosynthesis of this vitamin complex [32]. Different methods of parasite development control in the insect digestive tract, through paratransgenesis, have been proposed by several authors [33,34,35].

*S. marcescens*, a Gram-negative bacterium, belongs to the Enterobacteriaceae family. It is a ubiquitous bacterium found in different environments and the digestive tract of diverse animals [36] *S. marcescens* is found frequently in different species of triatomines collected both in the field and laboratory insectaries [21,24]. In *R. prolixus*, the *S. marcescens* strains isolated from the intestinal microbiota have in vitro trypanolytic activity against *T. cruzi* epimastigotes [19]. In addition, in vivo infections of *R. prolixus* with *T. cruzi* Dm28c strain reduce the expression of *S. marcescens* 16S rRNA in the anterior midgut [7,20]. *S. marcescens* is also found in the microbiota of other vector insects. The use of genetically modified *Serratia* has been proposed for the secretion of anti-plasmodium molecules in the mosquito *Anopheles*, as a paratransgenesis strategy to avoid parasite infection, preventing its transmission [37]. In addition to its beneficial effects on insects’ digestion and development, the intestinal microbiota is also essential in maintaining the homeostasis of the digestive tract immune response [16,38,39]. Vieira et al. [39] showed that Gram-negative *Escherichia coli* and Gram-positive *Staphylococcus aureus* oral infection induce differential antimicrobial peptide expression in the *R. prolixus* midgut [39]. Therefore, it is crucial to analyze the effects of *R. rhodnii* and *S. marcescens* (both bacteria naturally colonize the *R. prolixus* digestive tract), on *Rhodnius* immune response, in addition to their interactions with *T. cruzi*. 

In this context, the present work evaluated the modulation of *S. marcescens* and *R. rhodnii* on *R. prolixus* expression of antimicrobial peptides (AMPs), antimicrobial activity, phenoloxidase (PO) activity, and the production of reactive nitrogen species (RNS) through the expression of nitric oxide synthase (NOS). The insect immune responses modulated by each commensal bacteria could be used as a strategy to eliminate the parasite development in the insect. This may be the basis for the biotechnological development of bacteria focused on the expression of anti-parasitic agents, e.g., in the production of paratransgenic insects. Knowledge of relationships between triatomines and microbiota are of great importance in designing new control strategies for Chagas disease.

## 2. Results

### 2.1. Quantification of Serratia Marcescens and Rhodococcus Rhodnii by qPCR 

**Figure 1:**
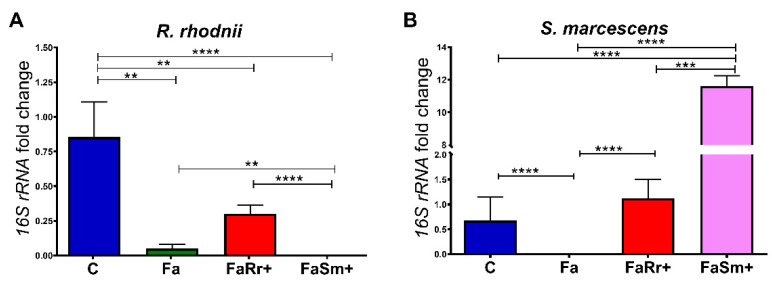
RT-qPCR determination of bacterial load in the anterior midgut of 5th instar nymphs of *Rhodnius prolixus* 7 days after feeding. The insects were previously treated with antibiotics as 4th instar nymphs and orally recolonized with *Rhodococcus rhodnii* or *Serratia marcescens* added to the blood meal of 5th instar nymphs. The antibiotic treatment consisted of ampicillin, penicillin, and hygromycin, with final concentrations of 150, 150, and 1 μg/mL, respectively, of defibrinated rabbit blood meal, and the treatment with *R. rhodnii* or *S. marcescens* added to the blood meal was 10^4^ and 10^3^ /mL, respectively. Analysis of relative expression of 16S-rRNAs gene from (**A**) *R. rhodnii* and (**B**) *S. marcescens* by RT-qPCR. Treatments: control (C); insects treated with antibiotics on 4th instar only (Fa); *R. rhodnii* (FaRr+); *S. marcescens* (FaSm+). Each bar represents the mean of relative quantification (RQ) values of 2 experiments, each experiment with 2 pools of 5 insect tissues each, corresponding to 20 insects (*n* = 4). The relative quantification by the ΔΔCt method was performed using the control group (insects fed with blood only) as the calibrator. All data were normalized to the *R. prolixus* 18S-rRNA. ΔΔCt values were analyzed by one-way ANOVA with the post-hoc Tukey test, ** *p* < 0.01; *** *p* < 0.001, **** *p* < 0.0001.

Treatment of 4th instar nymphs with antibiotics (group Fa, Figure 1) resulted in a significant decrease in the number of intestinal bacteria in the 5th instar nymphs after they received a regular blood supply when compared to the control group not treated with antibiotics (group C, Figure 1). This effect was observed for both intestinal bacteria analyzed, *R. rhodnii* (*p* < 0.01; Figure 1A), and *S. marcescens* (*p* < 0.0001; Figure 1B). 

Insects fed with *R. rhodnii* in the 5th instar, after the antibiotic treatment in the 4th instar (group FaRr+ in Figure 1A), had no significant difference in the amount of *R. rhodnii* compared to the nymphs only treated with antibiotic (group Fa in Figure 1A) or nymphs fed with *S. marcescens* (*p* < 0.0001; group FaSm+ in Figure 1A). However, the recolonization with *R. rhodnii* (group FaRr+, Figure 1A) did not result in similar levels to those of the control group, which was not treated with antibiotics (group C, Figure 1A). In the same experiment, recolonization with *R. rhodnii* resulted in amounts of *S. marcescens* (group FaRr+ in Figure 1B) that were comparable to controls not treated with antibiotics (*p* > 0.05; group C in Figure 1B), significantly higher than antibiotic-treated insects fed with blood only (*p* < 0.0001; group Fa in Figure 1B), and significantly lower than insects recolonized with *S. marcescens* after the antibiotics treatment (*p* < 0.001; group FaSm+ in Figure 1B). 

The 5th instar nymphs recolonized with *S. marcescens*, after treatment with antibiotics in the 4th instar, showed no detectable *R. rhodnii* counts in the 5th instar (group FaSm+ in Figure 1A). This result was significantly different than those of controls (*p* < 0.0001; group C in Figure 1A), insects treated with antibiotics (*p* < 0.01; group Fa in Figure 1A), and insects treated with antibiotics and fed with *R. rhodnii* (*p* < 0.0001; group FaRr+ in Figure 1A). As expected, this group of insects (FaSm+) orally recolonized with *S. marcescens* after treatment with antibiotics had significantly higher amounts of *S. marcescens* (group FaSm+ in Figure 1B) than insects treated with *R. rhodnii* (*p* < 0.001, group FaRr+ in Figure 1B), controls (*p* < 0.0001; group C in Figure 1B), and antibiotic-treated insects (*p* < 0.0001; group Fa in Figure 1B).

**Figure 2:**
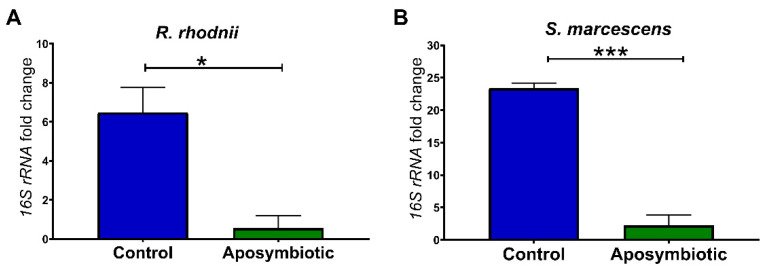
Determination of bacterial load in the anterior midgut of 1st instar nymphs of *Rhodnius prolixus* 5 days after feeding. Relative abundance of (**A**) *R. rhodnii 16S rRNA* (and (**B**) *S. marcescens 16S rRNA.* Treatments: Control group: eggs and insects kept in contact with adult feces; Aposymbiotic group: eggs treated with povidone-iodine 1% and maintained in sterile conditions. Each bar represents the mean of relative quantification (RQ) values of 2 experiments, each experiment with 2 pools of 5 insect tissues each, corresponding to 20 insects (*n* = 4). The relative quantification by the ΔΔCt method was performed using the relative abundance of *18S rRNA* of *R. prolixus* as the calibrator. ΔΔCt values were analyzed by an unpaired Student’s *t*-test, * *p* < 0.05; *** *p* < 0.001.

Another set of experiments was standardized using 1st instar aposymbiotic nymphs. Initially, we checked if these insects were free from contamination by *S. marcescens* and *R. rhodnii*. For this, the expression of *16S rRNA* genes of bacteria was also quantified. We observed that the aposymbiotic nymphs showed a reduction in the amount of *S. marcescens* (*p* < 0.001) and *R. rhodnii* (*p* < 0.05) in the anterior midgut when compared to control nymphs (Figure 2).

### 2.2. Phenoloxidase Enzyme Activity

**Figure 3:**
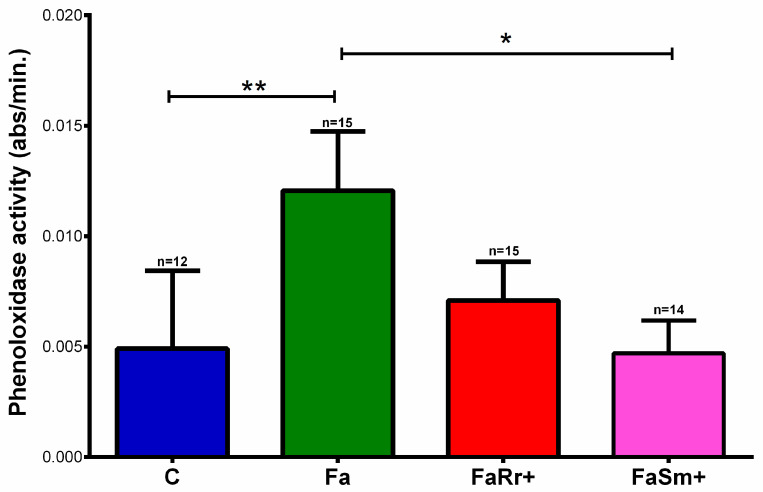
Phenoloxidase activity in the hemolymph of 5th instar nymphs of *Rhodnius prolixus* at 7 days after feeding. The treatments and group names are as described in Figure 1. Bars represent the mean ± SEM of three replicates of 5 insects; for each condition tested, n is represented above the bars. Means were analyzed by one-way ANOVA with the post-hoc Tukey test, * *p* < 0.05; ** *p* < 0.01.

After establishing the treatments with antibiotics and oral treatments with *R. rhodnii* and *S. marcescens*, we analyzed the PO activities in the hemolymph of the 5th instar nymph groups 7 days after feeding (DAF). The hemolymph PO activity in insects treated with antibiotics and fed with untreated blood (Fa) (*p* < 0.01) was higher than that in controls without antibiotics treatment (C) (Figure 3). The PO activity in the insects treated with antibiotics and recolonized with *R. rhodnii* (FaRr+) and *S. marcescens* (FaSm+) was similar to that for the controls (C).

### 2.3. Antibacterial Activity

**Figure 4:**
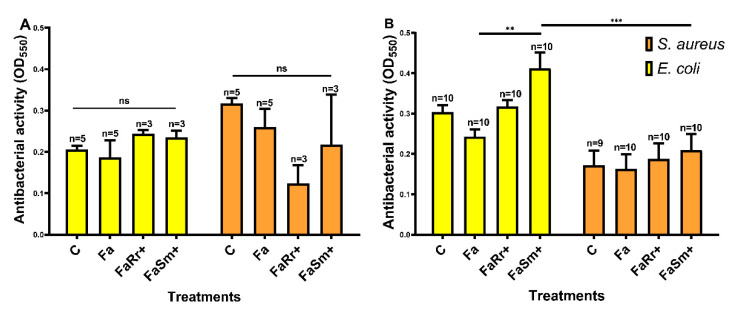
Antibacterial activity in the hemolymph and anterior midgut of 5th instar nymphs of *Rhodnius prolixus* 7 days after feeding. The treatments and group names are as described in Figure 1. The antibacterial activities were measured in vitro using (**A**) hemolymph and (**B**) anterior midgut, samples tested against *E. coli* (yellow bars) and *S. aureus* (orange bars) through the turbidimetric assay (OD_550 nm_) after 15 h incubation. Bars represent the mean ± SEM of two independent experiments with pools of 10 insects each; for each condition tested, n is represented above the bars. Means were compared between the different treatments (C, Fa, FaRr+ and FaSm+) and the different bacteria (*E. coli* and *S. aureus*) using two-way ANOVA with the post-hoc Tukey test; ** *p* < 0.01; *** *p* < 0.001; ns: not significant.

Two different biological fluids were assayed for antibacterial activity in 5th instar nymphs collected 7 DAF: the hemolymph and anterior midgut (Figure 4). These samples were tested against two different bacteria: *S. aureus*, a Gram-positive bacterium, and *E. coli*, a Gram-negative bacterium. In the hemolymph, no significant differences were observed in the antibacterial activities among the tested groups (Figure 4A).

In contrast, in the anterior midgut (Figure 4B), the insects recolonized with *S. marcescens* (FaSm+) had higher antibacterial activity against *E. coli* when compared to the antibiotic-treated group (Fa) (*p* < 0.01) and higher antibacterial activity against *E. coli* when compared with the same group (FaSm+) tested against *S. aureus* (*p* < 0.001).

### 2.4. Antimicrobial Peptides (AMP) Gene Expression

**Figure 5:**
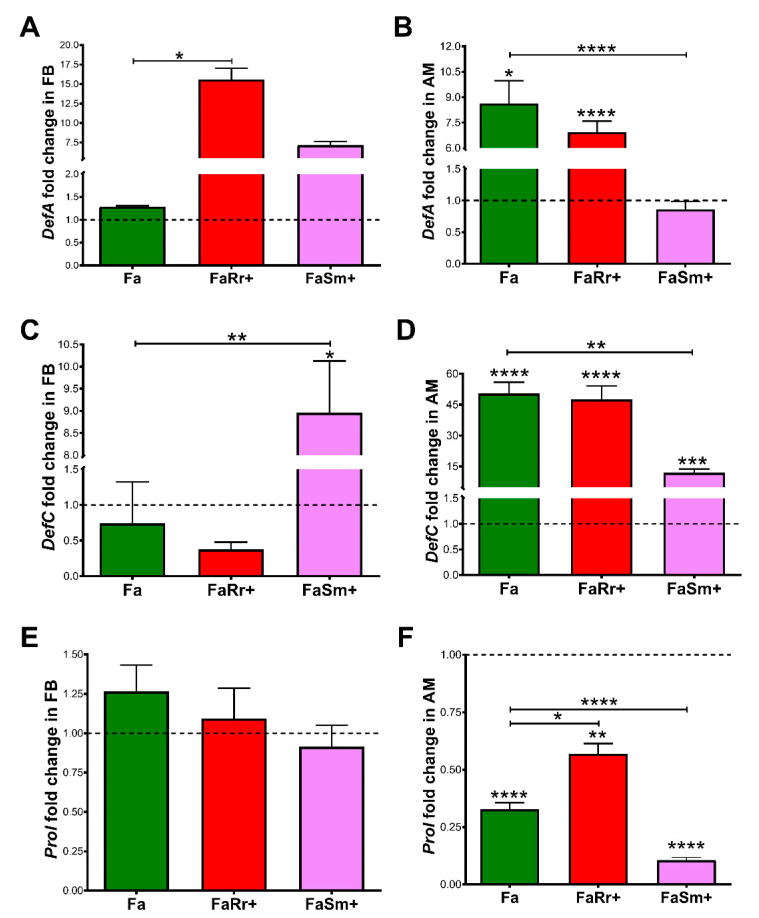
Antimicrobial peptide genes relative expression in the fat body (FB) and anterior midgut (AM) of *Rhodnius prolixus* 5th instar nymphs. The nymphs were previously fed with only antibiotic (Fa), *R. rhodnii* plus antibiotic (FaRr+), or *S. marcescens* plus antibiotic (FaSm+). Fat body (FB) and anterior midgut (AM) were collected 7 days after feeding. Relative expression of *DefA* (**A**,**B**), *DefC* (**C**,**D**), and *Prol* in (**E**,**F**) were analyzed in the fat body (**A**,**C**,**E**) and anterior midgut (**B**,**D**,**F**). Data were quantified using the gene expression of control insects as the calibrator. Bars represent the mean of 2 independent experiments—2 pools of 5 tissues each, with a total of 20 insects (*n* = 4). ΔΔCt values were analyzed by one-way ANOVA compared with the post-hoc Tukey test, * *p* < 0.05; ** *p* < 0.01; *** *p* < 0.001; **** *p* < 0.0001.

The 5th instar nymphs recolonized with *R. rhodnii* (FaRr+) had *DefA* expression levels in the fat body around 16-fold higher when compared to the group treated with antibiotics only (Fa) (*p* < 0.0 5, Figure 5A). Recolonization with *S. marcescens* (FaSm+) did not result in significant changes in *DefA* expression in the fat body (*p* > 0.05; Figure 5A). In the anterior midgut, the expression levels of *DefA* were significantly higher in the *R. rhodnii*-recolonized (FaRr+) (*p* < 0.0001) and antibiotic-treated (Fa) (*p* < 0.05) groups when compared to the controls (Figure 5B). The *S. marcescens* recolonized (FaSm+) group had similar *DefA* expression when compared to controls but significantly lower (*p* < 0.0001) when compared to the antibiotic-treated (Fa) group (Figure 5B).

Regarding the *DefC* gene, insects recolonized with *S. marcescens* (FaSm+) had higher expression levels in the fat body than the controls and the antibiotic-treated (Fa) groups (*p* < 0.05; Figure 5C). In the anterior midgut, the antibiotic-treated (Fa), *R. rhodnii* (FaRr+), and *S. marcescens* (FaSm+) recolonized groups had higher expressions levels of *DefC* when compared to the control group (*p* < 0.0001; Figure 5D). The *S. marcescens* (FaSm+)-recolonized group demonstrated lower *DefC* expression when compared to the antibiotic-treated group (Fa) (*p* < 0.01; Figure 5D).

There were no significant differences in the abundance of *Prol* transcripts between treatments in the fat body (*p* > 0.05; Figure 5E). In the anterior midgut, all treated groups had a lower relative expression of *Prol* when compared to controls (Fa, *p* < 0.0001; FaRr+, *p* < 0.01; FaSm+, *p* < 0.0001; Figure 5F). Insects of the group FaRr+ had higher expression of *Prol* than antibiotic-treated Fa (*p* < 0.05) insects. The FaSm+ group presented decreased *Prol* expression when compared to the antibiotic-treated group (Fa, *p* <0.0001; Figure 5F).

**Figure 6:**
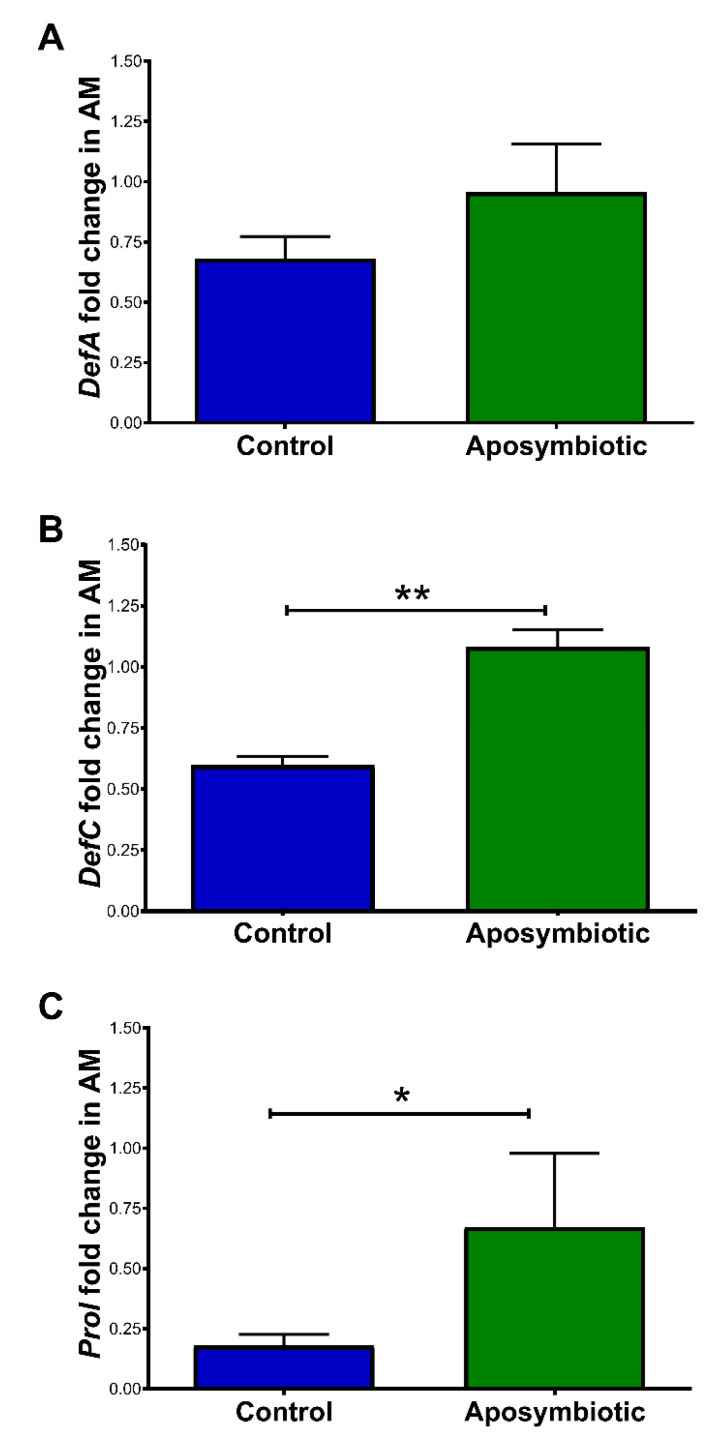
Relative expression of antimicrobial peptide genes in the anterior midgut (AM) of *Rhodnius prolixus* 1st instar nymphs 5 days after feeding. (**A**) *DefA*; **(B**) *DefC*; (**C**) *Prol*. Treatments: control group: eggs and insects were kept in contact with adult feces; aposymbiotic group: eggs previously treated with povidone-iodine 1% and maintained in sterile conditions. Data were quantified using the gene expression of control insects as the calibrator. Each bar represents the mean of relative quantification (RQ) values of 2 independent experiments—2 pools of 5 tissues each, with a total of 20 insects (*n* = 4). Bars represent the mean of 2 independent experiments—2 pools of 5 tissues each, with a total of 20 insects (*n* = 4). ΔΔCt values were analyzed by an unpaired Student’s *t*-test, * *p* < 0.05; ** *p* < 0.01.

In 1st instar nymphs, there were no significant differences in the expression of *DefA* in the anterior midgut when comparing aposymbiotic and control 1st instar nymphs (Figure 6A). Aposymbiotic nymphs showed an increase in the relative amounts of *DefC* (*p* < 0.01 Figure 6B) and *Prol* transcripts (*p* < 0.05; Figure 6C) when compared to the control group. 

### 2.5. Nitric Oxide Synthase (NOS) Gene Expression

**Figure 7:**
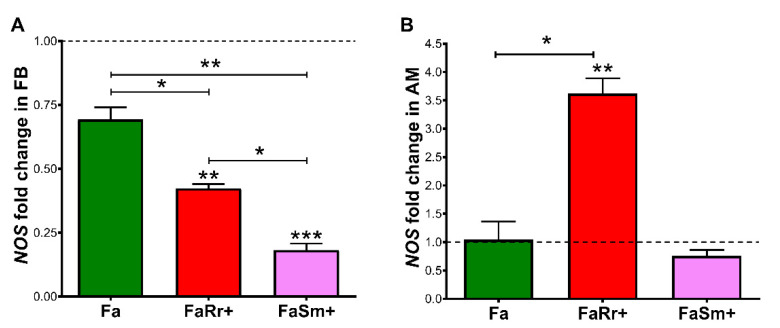
Nitric oxide synthase (*NOS*) relative expression in fat bod (FB) (**A**) and anterior midgut (AM) (**B**) of 5th instar nymphs of *Rhodnius prolixus* 7 days after feeding. The insects were previously treated with antibiotics as 4th instar nymphs and recolonized with *Rhodococcus rhodnii* or *Serratia marcescens* added to the blood meal of 5th instar nymphs. The treatments and group names are as described in Figure 1 Each bar represents the mean of relative quantification (RQ) values of 2 experiments, each with 2 pools of 5 insect tissues, with a total of 20 insects (*n* = 4). The relative quantification by the ΔΔCt method was performed using the control group (insects fed with blood only) as the calibrator. ΔΔCt values were analyzed by one-way ANOVA with the post-hoc Tukey test, * *p* < 0.05; ** *p* < 0.01; *** *p* < 0.001. The level of expression of the control group (arbitrarily set as 1) is shown with the dotted line. Asterisks above individual bars show significant differences to the controls, and asterisks above brackets show differences between two experimental groups.

Regarding *NOS* expression in the fat body insects recolonized with *R. rhodnii* (FaRr+) had significantly lower relative levels when compared to the control (*p* < 0.01) and antibiotic-treated (Fa) groups (*p* < 0.01; Figure 7A). Treatment with antibiotics (Fa) did not result in significant *NOS* expression changes compared to controls (Figure 7A). The FaSm+-infected group had lower levels of NOS when compared to the control group (*p* < 0.001), to the antibiotic-treated (Fa) group (*p* < 0.01), and to the *R. rhodnii*-recolonized (FaRr+) group (*p* < 0.05; Figure 7A).

In the anterior midgut, insects recolonized with *R. rhodnii* (FaRr+) had higher levels of *NOS* transcripts when compared to control groups and the group treated with antibiotics (Fa) (*p* < 0.01 and *p* < 0.05, respectively; Figure 7B).

## 3. Discussion

*Rhodnius prolixus* nymphs engage in coprophagy, which is essential for the acquisition of microorganisms from the other insects of their colony [40]. These ingested environmental microorganisms may contribute to triatomine development and protection against pathogenic microorganisms [13,41]. Herein, we observed the modulation of the *R. prolixus* immune system working with two described bacterial species associated with this triatomine, the symbiont *Rhodococcus rhodnii* and the generalist *Serratia marcescens*.

Initially, the insects were treated with a mixture of antibiotics that succeed to eliminate *R. rhodnii* and *S. marcescens* from the anterior midgut contents (Figure 1). We observed that the anterior midgut of the antibiotic-treated insects (Fa) presented higher expression of the AMPs *DefA* (8.6-fold) and *DefC* (50-fold), and reduced expression of *Prol* when compared with the control (Figure 5B,D,F). The aposymbiotic (egg surface-sterilized) 1st instar *R. prolixus* also presented higher expression of *DefC* and *Prol* in the anterior midgut in comparison to controls (Figure 6B,C). The upregulation of AMP genes was also observed in aposymbiotic hemiptera, *Dysdercus fasciatus* [42], and in larvae of *Drosophila* with nutrient deprivation [43]. 

It is known that the disturbance of the microbiota bacteria community by insect antibiotic treatment and diet diversity may cause metabolic and immunological imbalance [43,44,45,46]. Here, we observed that the pre-treatment with antibiotics also increased the PO activity in the hemolymph of *R. prolixus* (Figure 3). In contrast, the downregulation of PO activity in the *R. prolixus* midgut treated with antibiotics was observed in a previous publication [18]. However, the combination of antibiotics used was different and probably altered the microbiota composition in a different pattern. Furthermore, the tissues analyzed here and in [18] were different. The reduction of some bacteria from the insect gut microbiota could cause an imbalance in the proportion of microorganisms favoring the growth of other microorganism species, which are potentially pathogenic. This imbalance is known as dysbiosis; dysbiosis is associated with an intense immune stimulus in *Drosophila* [47], and could be the reason for the high PO activities and AMP expression observed in the 4th instar nymphs treated with antibiotics or 1st instar aposymbiotic nymphs. 

The recolonization of each commensal bacterium in the antibiotic-treated insects succeeds in growing the desired bacteria in the insect gut. Although the FaSm+ group was fed with antibiotics in the 4th instar and with *S. marcescens* in the 5th instar, there was an unexpected growth of *R. rhodnii* (Figure 1). We hypothesize that the presence of *S. marcescens* provides an indirect and unknown source of growth of other microorganisms, and this phenomenon needs to be better studied. Furthermore, we recognize that the experimental elimination of bacteria from the insect’s gut microbiota, followed by recolonization with only one commensal bacterium, may be limited due to several microbiota community effects that may not be captured by an experimental design. 

The overexpression of the antimicrobial peptide transcript *DefC* was also observed in the anterior midgut of *R. prolixus* treated with antibiotics and recolonized with *R. rhodnii* and *S. marcescens* (groups FaRr+ and FaSm+; Figure 5). Similarly, *DefC* expression was also enhanced in *R. prolixus* after the following challenges: injection into hemocoel of *Enterobacter cloacae* [48], and oral infection with *Escherichia coli* [39], *T. rangeli*, and *T. cruzi* [7,49]. In addition, some authors demonstrated that the expression of *DefC* was suppressed in *R. prolixus* after challenge with *S. aureus*, a Gram-positive bacterium, through injection and oral infection [39,48]. 

However, suppression of *DefC* expression appears to be more induced by Gram-negative bacteria and *Trypanosoma* spp. infection in *R. prolixus*, but additional roles of this AMP need further investigation. In the case of these trypanosomatids, the increase in the expression of *DefC* was associated with the reduction of the microbiota population [7]. The expression of *DefA* was also upregulated in *R. prolixus* recolonized with *R. rhodnii*, a Gram-positive bacterium, as in the case of *S. aureus* infection, which also increased *DefA* mRNA levels [39]. However, in the sand fly *Lutzomyia longipalpis*, oral feeding with different Gram-negative bacteria, such as *E. coli*, *Ochrobactrum* sp., and *S. marcescens* stimulated and increased defensin expression [50].

The AMP prolixicin, isolated initially from the *R. prolixus* fat body, has a higher in vitro effect on Gram-negative bacteria [51]. Vieira et al. [39] observed a downregulation of *Prol* transcript production in the anterior midgut of *R. prolixus* fed with *E. coli*. In agreement, downregulation of the *Prol* mRNA levels was observed in insects previously treated with antibiotics and in insects recolonized with *S. marcescens* (Figure 5). The low level of *Prol* transcripts was also observed in the control group of 1st instar nymphs that had a greater amount of *S. marcescens* than *R. rhodnii*, indicating that the effect is similar at different stages of development (Figure 6).

In addition to the higher expression of defensins A (Figure 5), higher PO activities were observed (Figure 3) in the hemolymph of antibiotic-treated and *R. rhodnii*-recolonized (FaRr+) groups when compared to controls. Although the bacteria of intestinal microbiota reside in the digestive tract, the microbiota reduction can interfere with the immune responses of the hemocoel in a systematic manner, as observed in the bean bug, *Riptortus pedestris* [52]. In this model, the insect line harboring the gut symbiont *Burkholderia* had higher humoral responses in the hemolymph when compared to the group lacking the gut symbiont [52]. In aposymbiotic tsetse flies (*Glossina morsitans*), reduced numbers of circulating and sessile hemocytes, and prophenoloxidase expression levels, were observed, making flies more susceptible to infection with the normally non-pathogenic *E. coli* [53]. 

Antibacterial activity occurs with the sum of humoral factors that prevent bacterial growth, which mainly relies on the activity of AMPs, but also on PO activity and the release of ROS and RNS [18,38,54]. It was previously observed that *R. prolixus* modulates antibacterial activity depending on the challenged bacterial species [39]. In the present work, the insects recolonized with *S. marcescens* presented higher antibacterial activity against the Gram-negative bacteria *E. coli* (Figure 4), which may be due to the effect of the increased defensin C in the anterior medium intestine also observed in these insects. In contrast, these insects presented lower antibacterial activity against the Gram-positive *S. aureus*. However, in *R. prolixus* infected with *S. aureus*, the anterior midgut contents had high antibacterial activity in vitro against the same bacteria, *S. aureus*, but not against *E. coli* [39], whereas insects infected with *E. coli* presented an increased antibacterial activity against *S. aureus* in the posterior midgut. Therefore, these antibacterial factors need to be further investigated.

The expression of *NOS* in the anterior midgut of insects recolonized with *R. rhodnii* demonstrated a four-fold increase when compared to controls. The excess of RNS produced by NOS causes a nitrosative stress that must be avoided, especially in the insect hemocoel. In *Anopheles gambiae*, the gut bacteria have genes responsible for regulating oxidative and nitrosative stress [55]. In previous work, our group observed a negative relationship between *NOS* expression and the development of *Trypanosoma cruzi* in the gut of *R. prolixus* [56]. This raises the question of whether *R. rhodnii* affects the development of trypanosomatids in the anterior midgut.

It is known that the intestinal microbiota can affect the vectorial competence of insect vectors in different ways, such as by competition for resources, by secretion of antipathogenic molecules, or by modulation of the insect’s immune response [57,58,59]. *A. gambiae* treated with antibiotics becomes more susceptible to infection by *Plasmodium falciparum* [60] and the infection of *A. gambiae* with *S. marcescens* demonstrates an anti-plasmodium effect [61].

This knowledge may lead to discovering new methods to block the transmission of pathogens, such as paratransgenesis. The use of *S. marcescens* in paratransgenesis to control *T. cruzi* transmission by triatomines could be considered. *S. marcescens* is found in several triatomine species captured in the field and the laboratory [21,24,49]. In addition, *S. marcescens* has also been proposed as a tool for controlling pathogen transmission by other insect vectors [62]. Moreover, bacteria such as *R. rhodnii* have proven to be a suitable candidate to be applied in paratransgenic approaches [33,35,40,63,64,65]. Here, we hypothesized that increased NOS gene expression in *R. prolixus* gut caused by *R. rhodnii* recolonization could be used as a strategy to prevent *T. cruzi* development in the vector since augmented NOS production in the host is related to a limiting factor for parasite infection. In conclusion, investigations about the mechanisms by which the gut microbiota interferes in vectorial competence are essential to find new targets for vector-borne disease insects.

## 4. Materials and Methods

### 4.1. Ethics Statement

Rabbit blood was provided by the *Instituto de Ciência e Tecnologia em Biomodelos* (ICTB/Fiocruz), which maintains and breeds animals following the Ethical Principles in Animal Experimentation. Blood collection was licensed and approved by *Comissão de Ética no Uso de Animais* from *Fundação Oswaldo Cruz* (CEUA/Fiocruz) under the protocol number L-019/17.

### 4.2. Insects Maintenance

*R. prolixus* was maintained in an insectary at *Laboratório de Bioquímica e Fisiologia de Insetos* (LABFISI) of *Instituto Oswaldo Cruz* (IOC) at the *Fundação Oswaldo Cruz* (Fiocruz), at 26–27 °C and 55–60% humidity. Insect feedings were regularly performed using defibrinated rabbit blood added to an artificial apparatus [66]. Only fully engorged insects were selected for the assays.

### 4.3. Bacteria Maintenance and Preparation for Recolonization 

*R. rhodnii* isolated from *R. prolixus insectary* from Swansea University was kindly provided by Prof. Norman Ratcliffe. A colony of *R. rhodnii* was inoculated in 10 mL of Tryptone soy broth (TSB) (Sigma-Aldrich, St. Louis, MO, USA) and maintained in an incubator at 30 °C, 90 rpm for 48 h before use for insect feeding. 

*S. marcescens* strain A1 was previously isolated from *R. prolixus* of the LABFISI insectary by Mota [20]. It was deposited in the *Coleção de Enterobacterias* (CENT) at the Fiocruz, Brazil. A colony of this bacteria was inoculated in 20 mL of TSB and maintained at 30 °C, 90 rpm for 18 h. Before using for insect recolonizations, bacteria were preserved at 4 °C for 24 h to diminish virulence. 

Both bacteria were washed in phosphate-buffered saline (PBS) (Sigma-Aldrich, St. Louis, MO, USA) (0.01 M phosphate buffer, 2.7 mM potassium chloride, and 0.137 M sodium chloride, pH 7.4) 2 times by centrifugation at 1890× *g* for 10 min at 4 °C, and the supernatant was removed. They were then suspended in PBS for a final concentration of 10^3^ and 10^4^ cells/mL and counted in a Neubauer chamber. All bacteria stocks were kept at −70 °C in brain–heart infusion (BHI) (Sigma-Aldrich, St. Louis, MO, USA) liquid media containing 10% (*v*/*v*) glycerol.

### 4.4. Insect Treatments and Recolonization

The commensal recolonization was done with two main bacteria, *S. marcescens and R. rhodnii*, encountered in the intestinal microbiota of *R. prolixus* from the insectary of LABFISI. *S. marcescens* is the most abundant species and *R. rhodnii* is the well-studied symbiont, both from *R. prolixus.* To clear the general bacteria from *R. prolixus* intestinal microbiota, the insects were treated with a combined antibiotic. Previous experiments with different antibiotic concentrations established the mixture of antibiotics capable of reducing the population of *S. marcescens* and *R. rhodnii* in the *R. prolixus* gut, causing minimal impact on mortality and ecdysis when compared to non-treated controls (Figure S1 and Figure 1). Therefore, the antibiotics standardized were ampicillin, penicillin and hygromycin (all purchased form Sigma-Aldrich, St. Louis, MO, USA), with respective final concentrations of 150, 150, and 1 μg/mL in defibrinated rabbit blood meal.

The 4th instar *R. prolixus* nymphs were starved for 30–40 days before treatments. Control groups (C) were fed on defibrinated rabbit blood at the 4th instar and subsequently after ecdysis, as were 5th instar nymphs. The blood containing the mixture of antibiotics was offered to a group of 4th instar nymphs which was, after ecdysis, subsequently fed blood alone or blood containing bacteria, as with 5th instar nymphs. Concentrations of bacteria given to the insects were selected for lack of impact on mortality and ecdysis (Figure S1). The *S. marcescens* or *R. rhodnii* were offered to the 5th instar nymphs previously treated with antibiotics at respective final concentrations of 1 × 10^3^ cells/mL and 1 × 10^4^ cells/mL, respectively, in defibrinated blood. Only fully engorged insects were selected after oral treatments. For the immune assays, insect samples were collected at 7th day after feeding (DAF) due to the known dynamic of bacterial growth [17] and greater intensity of immune response activation in 5th instar nymphs of *R. prolixus* after blood ingestion [7,18,67]. The scheme of treatments is summarized in Table 1.

### 4.5. Aposymbiotic Nymphs

To better understand the same events at another stage of development, the AMPs expression in the 1st instar stage was also observed. Adult *R. prolixus* females were separated for oviposition immediately after feeding. They were maintained in aseptic conditions for five days for egg collection. Glass vials used in aseptic conditions were autoclaved containing pieces of filter paper inside, and covered with a sterile cover made of cotton and gauze that protected the vial against contamination and allowed air passage. After being collected, the eggs were separated into two groups: aposymbiotic and control. The aposymbiotic group was obtained by sterilizing the eggs’ surface with commercial povidone (1% of active iodine *v*/*v*) for 20 min and washing three times with sterile water [63]. The eggs treatment with commercial povidone does not affect egg hatching time, nor nymph viability. Then, eggs were maintained in the same sterile conditions as described for oviposition. Control eggs were washed in water and kept in non-sterile conditions inside a glass with filter paper impregnated with adult feces from the insectary. 

Two weeks later, the 1st instar nymphs, from sterilized and control eggs, were fed inside the laminar flow cabinet, using autoclaved artificial feeders as described before, in sterile conditions.

### 4.6. Antimicrobial Peptides (AMPs) and NOS Gene Expression, and Quantification of Serratia Marcescens and Rhodococcus Rhodnii by qPCR

The 5th instar nymphs recolonized with bacteria at the 7th DAF were dissected to obtain two pools of five fat bodies and anterior midgut samples each. In addition, 1st instar nymphs, and aposymbiotic and control nymphs, were dissected 5 DAF to obtain the anterior midguts, which were separated into two pools of five tissue samples each [39]. Total RNA was extracted using a NucleoSpin^®^ RNA II Kit (Macherey-Nagel, Düren, Germany) using the manufacturer’s instructions, and quantified using a NanoDrop 2000 Spectrophotometer^®^ (Thermo Scientific, Waltham, MA, USA). cDNA was synthesized with a First-Strand cDNA Synthesis Kit^®^ (GE Healthcare, Buckinghamshire, UK) following the manufacturer’s protocol using 2.5 µg of total RNA and pd(N)_6_ primer (Table S1). The cDNA obtained was quantified by fluorescence using a Qubit Fluorimeter (Life Technologies) with the ssDNA assay kit. Real-time quantitative polymerase chain reactions (qPCR) were performed in an ABI PRISM 7500 Sequence Detection System^®^ (Applied Biosystems) at the PDTIS/Fiocruz facilities. 

Each measurement was made in triplicate for each pool of insects (two pools of five tissues; *n* = 2, representing 10 insects). Each reaction contained 10 ng cDNA, primers (0.25 μM), and the GoTaq qPCR master mix (Promega) in a final volume of 20 μL. Reactions were incubated at 95 °C for 10 min, followed by 40 cycles of 95 °C for 15 s and 60 °C for 1 min. As negative controls, reactions were carried out without the cDNA template. Melting curve analysis was carried out to confirm that only a single product was amplified for each target. Primers used are described in Supplementary Table S1. The AMPs analyzed herein was defensins A and C and prolixicin. Defensin B was not investigated here, since as previously seen, DefB is significantly downregulated during parasite infection, difficulting gene expression assessment by RT-qPCR due primer dimer formation [7]. The AMPs and NOS gene expression in the tissues of *R. prolixus* were quantified by the comparative Ct (ΔΔCt) method [68], using GAPDH, α-tubulin and 18S-rRNA as *R. prolixus* housekeeping genes. Data were analyzed by the Expression Suite v1.0.3 software (Life Technologies), considering the amplification efficiency of each target.

### 4.7. Antibacterial Activity

To analyze the antibacterial activity in the hemolymph and anterior midgut, we used the turbidimetric assay as previously described [39]. 

Dissections of 5th instar nymphs were performed on 7th DAF. Hemolymph was collected from the insects after sanitizing the cuticle with 70% alcohol. Then, the forelegs were cut off and the hemolymph with free-circulating hemocytes was collected by pipetting. Three pools with 10 insects each were used, obtained in 2 different experiments (*n* = 6, representing 60 insects) and diluted 1:1 in ultrapure water in sterile 1.5 mL tubes containing 1.5 µL of a saturated solution of phenylthiourea to avoid melanization. The anterior midgut was homogenized in 200 µL of PBS and centrifuged at 10,000× *g* for 1 min at 4 °C. Aliquots of 70 µL of the supernatant were transferred into tubes containing 630 µL of ultrapure water. All samples were filtered in a sterile 0.22 µm filter and maintained in sterile conditions, frozen at −20 °C until the assays. 

For these assays, we used *Escherichia coli* (K12 4401) and *Staphylococcus aureus* (9518), both obtained from the National Collection of Industrial and Marine Bacteria (NCIMB), Aberdeen, UK. Cultures of bacteria in exponential growth were washed in PBS as previously described [39] and diluted in tryptone soy broth (TSB) to a final concentration of 1 × 10^4^ cells/mL (90 uL sample + 10 uL of bacterial culture in a concentration of 1 × 10^5^ cells/mL). Controls were performed with the growth of the same concentrations of bacteria in peptone.

The bacterial growth was measured at 550 nm (OD_550_) during a 15 h incubation at 37 °C, with readings at hourly intervals in a microplate reader (SpectraMax 190, Molecular Devices). Data points were blanked against time zero, and the readings of the control wells were subtracted from all sample readings. The antibacterial activity is the difference between the readings of the bacteria growth in the control wells and the readings of bacteria growth in the sampled wells.

### 4.8. Phenoloxidase (PO) Assay

For PO activity measurement, the hemolymph was collected as described above in three replicates of 5 insects (*n* = 15). Samples were collected 7 DAF and diluted ten times in 10 mM sodium cacodylate buffer pH 7.4. The method was performed as described by Genta et al. [67].

The assay was prepared in triplicate by incubating 10 μL of the sample with 35 μL of 10 mM sodium cacodylate buffer pH 7.4 and 25 μL of a saturated solution of L-DOPA (4 mg/mL in sodium cacodylate buffer). The absorbance at 490 nm was measured for 120 min at 37 °C, with readings taken every 15 min in a microplate reader (SpectraMax 190, Molecular Devices). The values of enzymatic activity are expressed as abs/min.

### 4.9. Statistical Analyses

Statistical analysis was performed using GraphPad Prism 8.0.2 (San Diego, CA, USA). The D’Agostino–Pearson omnibus K2 normality test was used for verification of Gaussian distributions. For comparison of normally distributed data, an unpaired Student’s *t*-test or one-way ANOVA or two-way ANOVA was used depending of the numbers of groups tested, followed by Tukey’s multiple comparison tests. The F-test was used to check the equality of variances between samples. Differences among groups were considered statistically significant when *p* < 0.05. Results are reported as mean ± error (SEM). Probability levels are specified in the text and figure legends. For survival analysis, a Kaplan–Meier plot was used.

## Figures and Tables

**Table 1:** The protocol designed to treat the different groups of *Rhodnius prolixus* nymphs.

Insect Groups	4th Instar	5th Instar
	Antibiotic Treatment ^†^	Bacteria Recolonization
Control (C)	_	_
Antibiotics (Fa)	+	_
*S. marcescens* + antibiotics (FaSm+) ^††^	+	+
*R. rhodnii* + antibiotics (FaRr+) ^†††^	+	+

^†^ A mixture of antibiotics was added to the blood meal at final concentration of ampicillin (150 μg/mL), penicillin (150 μg/mL), and hygromycin (1 μg/mL). ^††^ *S. marcescens* (FaSm+) was added to the blood meal in final concentration of 1 × 10^3^ cells/mL. ^†††^ *R. rhodnii* (FaRr+) was added to the blood meal in final concentration of 1 × 10^4^ cells/mL.

## Supplementary Materials

The following are available online at https://www.mdpi.com/article/10.3390/ijms222010901/s1.

## References

[1] Chagas C. (1909). Nova tripanozomiaze humana: Estudos sobre a morfologia e o ciclo evolutivo do *Schizotrypanum cruzi* n. gen., n. sp., agente etiologico de nova entidade morbida do homem. Memórias Inst. Oswaldo Cruz.

[2] WHO Chagas Disease (American Trypanosomiasis). https://www.who.int/chagas/epidemiology/en/.

[3] Costa J., Peterson A.T. (2012). Ecological niche modeling as a tool for understanding distributions and interactions of vectors, hosts, and etiologic agents of Chagas disease. Adv. Exp. Med. Biol..

[4] Coura J.R. (2015). The main sceneries of Chagas disease transmission. The vectors, blood and oral transmissions—A comprehensive review. Memórias Inst. Oswaldo Cruz.

[5] Garcia E.S., Castro D.P., Figueiredo M.B., Azambuja P. (2012). Parasite-mediated interactions within the insect vector: *Trypanosoma rangeli* strategies. Parasites Vectors.

[6] Azambuja P., Garcia E.S., Ratcliffe N.A. (2005). Gut microbiota and parasite transmission by insect vectors. Trends Parasitol..

[7] Vieira C.S., Waniek P.J., Castro D.P., Mattos D.P., Moreira O.C., Azambuja P. (2016). Impact of *Trypanosoma cruzi* on antimicrobial peptide gene expression and activity in the fat body and midgut of *Rhodnius prolixus*. Parasites Vectors.

[8] Kollien A.H., Schaub G.A. (2000). The development of *Trypanosoma cruzi* in triatominae. Parasitol. Today.

[9] Nogueira N.P., Saraiva F.M., Sultano P.E., Cunha P.R., Laranja G.A., Justo G.A., Sabino K.C., Coelho M.G., Rossini A., Atella G.C. (2015). Proliferation and differentiation of *Trypanosoma cruzi* inside its vector have a new trigger: Redox status. PLoS ONE.

[10] Shikanai-Yasuda M.A., Marcondes C.B., Guedes L.A., Siqueira G.S., Barone A.A., Dias J.C., Amato Neto V., Tolezano J.E., Peres B.A., Arruda Júnior E.R. (1991). Possible oral transmission of acute Chagas’ disease in Brazil. Rev. Inst. Med. Trop. Sao Paulo.

[11] Futo M., Armitage S.A., Kurtz J. (2015). Microbiota Plays a Role in Oral Immune Priming in *Tribolium castaneum*. Front. Microbiol..

[12] Morella N.M., Koskella B. (2017). The Value of a Comparative Approach to Understand the Complex Interplay between Microbiota and Host Immunity. Front. Immunol..

[13] Salcedo-Porras N., Umaña-Diaz C., Bitencourt R.O.B., Lowenberger C. (2020). The Role of Bacterial Symbionts in Triatomines: An Evolutionary Perspective. Microorganisms.

[14] Douglas A.E. (2015). Multiorganismal insects: Diversity and function of resident microorganisms. Annu. Rev. Entomol..

[15] Habineza P., Muhammad A., Ji T., Xiao R., Yin X., Hou Y., Shi Z. (2019). The Promoting Effect of Gut Microbiota on Growth and Development of Red Palm Weevil, *Rhynchophorus ferrugineus* (Olivier) (Coleoptera: Dryophthoridae) by Modulating Its Nutritional Metabolism. Front. Microbiol..

[16] Muhammad A., Habineza P., Ji T., Hou Y., Shi Z. (2019). Intestinal Microbiota Confer Protection by Priming the Immune System of Red Palm Weevil *Rhynchophorus ferrugineus* Olivier (Coleoptera: Dryophthoridae). Front. Physiol..

[17] Azambuja P., Feder D., Garcia E.S. (2004). Isolation of *Serratia marcescens* in the midgut of *Rhodnius prolixus*: Impact on the establishment of the parasite *Trypanosoma cruzi* in the vector. Exp. Parasitol..

[18] Castro D.P., Moraes C.S., Gonzalez M.S., Ratcliffe N.A., Azambuja P., Garcia E.S. (2012). *Trypanosoma cruzi* immune response modulation decreases microbiota in *Rhodnius prolixus* gut and is crucial for parasite survival and development. PLoS ONE.

[19] Castro D.P., Seabra S.H., Garcia E.S., de Souza W., Azambuja P. (2007). *Trypanosoma cruzi*: Ultrastructural studies of adhesion, lysis and biofilm formation by *Serratia marcescens*. Exp. Parasitol..

[20] da Mota F.F., Castro D.P., Vieira C.S., Gumiel M., de Albuquerque J.P., Carels N., Azambuja P. (2018). In vitro Trypanocidal Activity, Genomic Analysis of Isolates, and in vivo Transcription of Type VI Secretion System of *Serratia marcescens* Belonging to the Microbiota of *Rhodnius prolixus* Digestive Tract. Front. Microbiol..

[21] da Mota F.F., Marinho L.P., Moreira C.J., Lima M.M., Mello C.B., Garcia E.S., Carels N., Azambuja P. (2012). Cultivation-independent methods reveal differences among bacterial gut microbiota in triatomine vectors of Chagas disease. PLoS Negl. Trop. Dis..

[22] Dias F.A., Gandara A.C., Perdomo H.D., Gonçalves R.S., Oliveira C.R., Oliveira R.L., Citelli M., Polycarpo C.R., Santesmasses D., Mariotti M. (2016). Identification of a selenium-dependent glutathione peroxidase in the blood-sucking insect *Rhodnius prolixus*. Insect Biochem. Mol. Biol..

[23] Eichler S., Schaub G.A. (2002). Development of symbionts in triatomine bugs and the effects of infections with trypanosomatids. Exp. Parasitol..

[24] Gumiel M., da Mota F.F., Rizzo Vde S., Sarquis O., de Castro D.P., Lima M.M., Garcia Ede S., Carels N., Azambuja P. (2015). Characterization of the microbiota in the guts of *Triatoma brasiliensis* and *Triatoma pseudomaculata* infected by *Trypanosoma cruzi* in natural conditions using culture independent methods. Parasit. Vectors.

[25] Duncan J.T. (1926). On a Bactericidal Principle present in the Alimentary Canal of Insects and Arac. Parasitology.

[26] Wigglesworth V.B. (1936). symbiotic bacteria in a blood-sucking insect, *Rhodnius prolixus* stal. (hemiptera, triatomidae). Parasitology.

[27] Auden D.T. (1974). Studies on the development of *Rhodnius prolixus* and the effects of its symbiote Nocardia rhodnii. J. Med. EntoMol..

[28] Baines S. (1956). The role of the symbiotic bacteria in the nutrition of *Rhodnius prolixus* (hemiptera). Exp. Biol..

[29] Ben-Yakir D. (1987). Growth retardation of *Rhodnius prolixus* symbionts by immunizing host against *Nocardia* (*Rhodococcus*) *rhodnii*. J. Insect Physiol..

[30] Lake P., Friend W.G. (1968). The use of artificial diets to determine some of the effects of *Nocardia rhodnii* on the development of *Rhodnius prolixus*. J. Insect Physiol..

[31] Pachebat J.A., van Keulen G., Whitten M.M., Girdwood S., Del Sol R., Dyson P.J., Facey P.D. (2013). Draft Genome Sequence of *Rhodococcus rhodnii* Strain LMG5362, a Symbiont of *Rhodnius prolixus* (Hemiptera, Reduviidae, Triatominae), the Principle Vector of *Trypanosoma cruzi*. Genome Announc..

[32] Tobias N.J., Eberhard F.E., Guarneri A.A. (2020). Enzymatic biosynthesis of B-complex vitamins is supplied by diverse microbiota in the *Rhodnius prolixus* anterior midgut following *Trypanosoma cruzi* infection. Comput. Struct. Biotechnol. J..

[33] Durvasula R.V., Gumbs A., Panackal A., Kruglov O., Taneja J., Kang A.S., Cordon-Rosales C., Richards F.F., Whitham R.G., Beard C.B. (1999). Expression of a functional antibody fragment in the gut of *Rhodnius prolixus* via transgenic bacterial symbiont *Rhodococcus rhodnii*. Med. Vet. EntoMol..

[34] Jose C., Klein N., Wyss S., Fieck A., Hurwitz I., Durvasula R. (2013). Recombinant Arthrobacter β-1, 3-glucanase as a potential effector molecule for paratransgenic control of Chagas disease. Parasit. Vectors.

[35] Taracena M.L., Oliveira P.L., Almendares O., Umaña C., Lowenberger C., Dotson E.M., Paiva-Silva G.O., Pennington P.M. (2015). Genetically modifying the insect gut microbiota to control Chagas disease vectors through systemic RNAi. PLoS Negl. Trop. Dis..

[36] Petersen L.M., Tisa L.S. (2013). Friend or foe? A review of the mechanisms that drive *Serratia* towards diverse lifestyles. Can. J. Microbiol..

[37] Wang S., Dos-Santos A.L.A., Huang W., Liu K.C., Oshaghi M.A., Wei G., Agre P., Jacobs-Lorena M. (2017). Driving mosquito refractoriness to *Plasmodium falciparum* with engineered symbiotic bacteria. Science.

[38] Garcia E.S., Castro D.P., Figueiredo M.B., Azambuja P. (2010). Immune homeostasis to microorganisms in the guts of triatomines (Reduviidae)—A review. Mem. Inst. Oswaldo Cruz.

[39] Vieira C.S., Waniek P.J., Mattos D.P., Castro D.P., Mello C.B., Ratcliffe N.A., Garcia E.S., Azambuja P. (2014). Humoral responses in *Rhodnius prolixus*: Bacterial feeding induces differential patterns of antibacterial activity and enhances mRNA levels of antimicrobial peptides in the midgut. Parasit. Vectors.

[40] Beard C.B., Durvasula R.V., Richards F.F. (1998). Bacterial symbiosis in arthropods and the control of disease transmission. Emerg. Infect Dis..

[41] Harington J.S. (1960). Studies on *Rhodnius prolixus*: Growth and development of normal and sterile bugs, and the symbiotic relationship. Parasitology.

[42] Bauer E., Salem H., Marz M., Vogel H., Kaltenpoth M. (2014). Transcriptomic immune response of the cotton stainer *Dysdercus fasciatus* to experimental elimination of vitamin-supplementing intestinal symbionts. PLoS ONE.

[43] Becker T., Loch G., Beyer M., Zinke I., Aschenbrenner A.C., Carrera P., Inhester T., Schultze J.L., Hoch M. (2010). FOXO-dependent regulation of innate immune homeostasis. Nature.

[44] Flatt T., Heyland A., Rus F., Porpiglia E., Sherlock C., Yamamoto R., Garbuzov A., Palli S.R., Tatar M., Silverman N. (2008). Hormonal regulation of the humoral innate immune response in *Drosophila melanogaster*. J. Exp. Biol..

[45] Garrett W.S., Gordon J.I., Glimcher L.H. (2010). Homeostasis and inflammation in the intestine. Cell.

[46] Krams I.A., Kecko S., Jõers P., Trakimas G., Elferts D., Krams R., Luoto S., Rantala M.J., Inashkina I., Gudrā D. (2017). Microbiome symbionts and diet diversity incur costs on the immune system of insect larvae. J. Exp. Biol..

[47] Chen K., Luan X., Liu Q., Wang J., Chang X., Snijders A.M., Mao J.H., Secombe J., Dan Z., Chen J.H. (2019). Drosophila Histone Demethylase KDM5 Regulates Social Behavior through Immune Control and Gut Microbiota Maintenance. Cell Host Microbe.

[48] Salcedo-Porras N., Guarneri A., Oliveira P.L., Lowenberger C. (2019). *Rhodnius prolixus*: Identification of missing components of the IMD immune signaling pathway and functional characterization of its role in eliminating bacteria. PLoS ONE.

[49] Vieira C.S., Mattos D.P., Waniek P.J., Santangelo J.M., Figueiredo M.B., Gumiel M., da Mota F.F., Castro D.P., Garcia E.S., Azambuja P. (2015). *Rhodnius prolixus* interaction with *Trypanosoma rangeli*: Modulation of the immune system and microbiota population. Parasit. Vectors.

[50] Telleria E.L., Sant’Anna M.R., Alkurbi M.O., Pitaluga A.N., Dillon R.J., Traub-Csekö Y.M. (2013). Bacterial feeding, *Leishmania infection* and distinct infection routes induce differential defensin expression in *Lutzomyia longipalpis*. Parasit. Vectors.

[51] Ursic-Bedoya R., Buchhop J., Joy J.B., Durvasula R., Lowenberger C. (2011). Prolixicin: A novel antimicrobial peptide isolated from *Rhodnius prolixus* with differential activity against bacteria and *Trypanosoma cruzi*. Insect Mol. Biol..

[52] Kim J.K., Lee J.B., Huh Y.R., Jang H.A., Kim C.H., Yoo J.W., Lee B.L. (2015). *Burkholderia* gut symbionts enhance the innate immunity of host *Riptortus pedestris*. Dev. Comp. Immunol..

[53] Weiss B., Aksoy S. (2011). Microbiome influences on insect host vector competence. Trends Parasitol..

[54] Whitten M., Sun F., Tew I., Schaub G., Soukou C., Nappi A., Ratcliffe N. (2007). Differential modulation of *Rhodnius prolixus* nitric oxide activities following challenge with *Trypanosoma rangeli*, *T. cruzi* and bacterial cell wall components. Insect Biochem. Mol. Biol..

[55] Wang Y., Gilbreath T.M., Kukutla P., Yan G., Xu J. (2011). Dynamic gut microbiome across life history of the malaria mosquito *Anopheles gambiae* in Kenya. PLoS ONE.

[56] Batista K., Vieira C.S., Florentino E.B., Caruso K.F.B., Teixeira P.T.P., Moraes C.D.S., Genta F.A., de Azambuja P., de Castro D.P. (2020). Nitric oxide effects on *Rhodnius prolixus*’s immune responses, gut microbiota and *Trypanosoma cruzi* development. J. Insect Physiol..

[57] Dennison N.J., Jupatanakul N., Dimopoulos G. (2014). The mosquito microbiota influences vector competence for human pathogens. Curr. Opin. Insect Sci..

[58] Douglas A.E. (2014). The molecular basis of bacterial-insect symbiosis. J. Mol. Biol..

[59] Ricci I., Valzano M., Ulissi U., Epis S., Cappelli A., Favia G. (2012). Symbiotic control of mosquito borne disease. Pathog. Glob. Health.

[60] Dong Y., Manfredini F., Dimopoulos G. (2009). Implication of the mosquito midgut microbiota in the defense against malaria parasites. PLoS Pathog..

[61] Bahia A.C., Dong Y., Blumberg B.J., Mlambo G., Tripathi A., BenMarzouk-Hidalgo O.J., Chandra R., Dimopoulos G. (2014). Exploring *Anopheles* gut bacteria for *Plasmodium* blocking activity. Environ. Microbiol..

[62] Huang W., Wang S., Jacobs-Lorena M. (2020). Use of Microbiota to Fight Mosquito-Borne Disease. Front. Genet..

[63] Dotson E.M., Plikaytis B., Shinnick T.M., Durvasula R.V., Beard C.B. (2003). Transformation of *Rhodococcus rhodnii*, a symbiont of the Chagas disease vector *Rhodnius prolixus*, with integrative elements of the L1 mycobacteriophage. Infect Genet. Evol..

[64] Fieck A., Hurwitz I., Kang A.S., Durvasula R. (2010). *Trypanosoma cruzi*: Synergistic cytotoxicity of multiple amphipathic anti-microbial peptides to *T. cruzi* and potential bacterial hosts. Exp. Parasitol..

[65] Matthews S., Rao V.S., Durvasula R.V. (2011). Modeling horizontal gene transfer (HGT) in the gut of the Chagas disease vector *Rhodnius prolixus*. Parasit. Vectors.

[66] Azambuja P., Garcia E.S., Crampton J., Beard C., Louis C. (1997). Care and maintenance of triatomine colonies. Molecular Biology of Insect Disease Vectors: A Methods Manual.

[67] Genta F.A., Souza R.S., Garcia E.S., Azambuja P. (2010). Phenol oxidases from *Rhodnius prolixus*: Temporal and tissue expression pattern and regulation by ecdysone. J. Insect Physiol..

[68] Livak K.J., Schmittgen T.D. (2001). Analysis of relative gene expression data using real-time quantitative PCR and the 2(-ΔΔC(T)) Method. Methods.

