# Supplementary material for: Influence of Serratia marcescens and Rhodococcus rhodnii on the Humoral Immunity of Rhodnius prolixus
Source: Int J Mol Sci. 2021 Oct 9;22(20):10901. doi: 10.3390/ijms222010901 (PMC8536199; doi:10.3390/ijms222010901)
Supplement: Supplementary file 1 [file ijms-22-10901-s001.zip › ijms-1313006-supplementary.pdf]

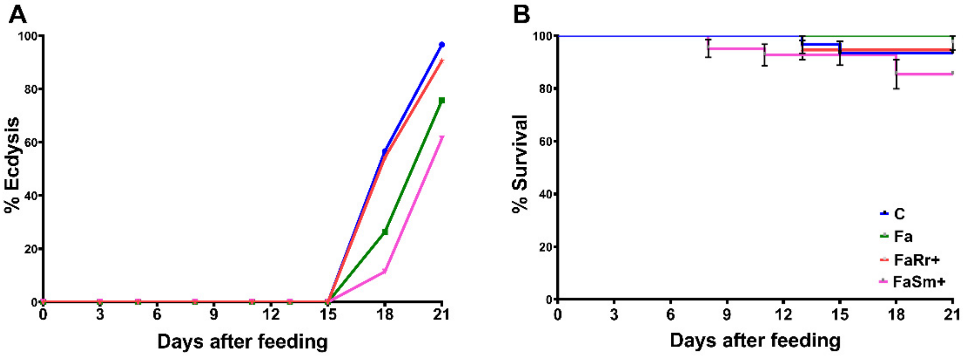

Figure S1. Survival and molting of 5th instar nymphs of *Rhodnius prolixus* recolonized with *Rhodococcus rhodnii* or *Serratia marcescens*. Ecdysis (**A**) and survival (**B**). The insects were previously treated with antibiotics as nymphs of the 4th instar, except for the control group and, after molting, recolonized with *Rhodococcus rhodnii* or *Serratia marcescens* by adding the bacteria to the blood feeding. Antibiotics treatment of 4th instar consisted of ampicillin, penicillin, and hygromycin with final concentrations of 150, 150, and 1 μg/mL, respectively, added in defibrinated rabbit blood. For recolonization with *R. rhodnii* or *S. marcescens*, a concentration of 10^3 and 10^4 cells/mL was added to the blood meal, respectively. Legend: control (C, in blue); antibiotic control (A in green); *R. rhodnii* (FaRr+, in red); *S. marcescens* (FaSm+ in pink). Percentage of 2 experiments using for each group 3 pools containing 10 insects each, *n* = 6, representing 60 insects. The log-rank (Mantel–Cox) test was performed for survival statistics analysis, ns.

Table S1. Primers used in RT-qPCR experiments and additional information.

Primers	Sequence (5′-3′)	GenBank Accession Number	Amplicon Size (pb)	References
α-tubulin-F *	TTTCCTCGATCACTGCTTCC	ACPB02030650	129	[1]
α-tubulin-R *	CGGAAATAACTGGGGCATAA			
GAPDH-F *	GATGGCGCCCAGTACATAGT	ACPB02038754	111	[1]
GAPDH-R *	AGCTGACGGGGCTGTTATTA			
18S-R.prolixus-F *	TCCTTCGTGCTAGGAATTGG		105	[1]
18S-R.prolixus-R *	GTACAAAGGGCAGGGACGTA			[1]
DefA-F	GAATACTCCACTCAACCGCAAC	AY196130	295	[2]
DefA-R	TAGTTCCTTTACATCGGCCA			
DefC-F	CAGTACAGTCCTAATACCTAGCC	AY196132	300	[2]
DefC-R	CAGTTCCTACGCAACGGCCT			
Prol-F	CTATAACGAGTGAACTATAAGACAA	EU448993	406	[2]
Prol-R	GTGTTTAATGGCGGTAACAAATTAC			
NOS-F	AATG GGCACCAGAAGTGTTC	U59389	238	[3]
NOS-R	GTTGCCGATTCCACAAATCT			
16S-S.Marcescens-F	GGTGAGCTTAATACGTTCATCAATTG	AJ233431	179	[4]
16S-S.Marcescens-R	GCAGTTCCCAGGTTGAGCC			
16S-R.rhodnii-F	CACTGGTTGCATGGCCTGGTG	EU650780	418	[2]
16S-R.rhodnii-R	TGAGCTGTGGGATTTCACAGAC			

* Reference genes.

References

1. Paim, R.M.M.; Araujo, R.N.; Lehane, M.J.; Gontijo, N.F.; Pereira, M.H. Application of RNA interference in triatomine (Hemiptera: Reduviidae) studies. *Insect Sci.* **2012**, *20*, 40–52, doi:10.1111/j.1744-7917.2012.01540.x.
2. Vieira, C.S.; Waniek, P.J.; Mattos, D.P.; Castro, D.P.; Mello, C.B.; Ratcliffe, N.A.; Garcia, E.S.; Azambuja, P. Humoral responses in *Rhodnius prolixus*: Bacterial feeding induces differential patterns of antibacterial activity and enhances mRNA levels of antimicrobial peptides in the midgut. *Parasit. Vectors* **2014**, *7*, 232, doi:10.1186/1756-3305-7-232.
3. Batista, K.; Vieira, C.S.; Florentino, E.B.; Caruso, K.F.B.; Teixeira, P.T.P.; Moraes, C.D.S.; Genta, F.A.; de Azambuja, P.; de Castro, D.P. Nitric oxide effects on *Rhodnius prolixus*'s immune responses, gut microbiota and *Trypanosoma cruzi* development. *J. Insect Physiol.* **2020**, *126*, 104100, doi:10.1016/j.jinsphys.2020.104100.

4. Saikaly, P.E.; Barlaz, M.A.; de Los Reyes, F.L., 3rd. Development of quantitative real-time PCR assays for detection and quantification of surrogate biological warfare agents in building debris and leachate. *Appl. Environ. Microbiol.* **2007**, *73*, 6557–6565, doi:10.1128/AEM.00779-07.
